# PAA, a novel metal chelator and metalloproteinase inhibitor significantly reduced amyloid plaque burden in an APP/tau mouse model of Alzheimer’s disease (AD)

**DOI:** 10.1002/alz.090979

**Published:** 2025-01-09

**Authors:** Larry Schmued, Calvert Schmued, Bryan Maloney, Debomoy K. Lahiri

**Affiliations:** ^1^ Histo‐Chem Inc, White Hall, AR USA; ^2^ Indiana University School of Medicine, Department of Psychiatry, Indianapolis, IN USA; ^3^ Indiana Alzheimer’s Disease Research Center, Indianapolis, IN USA

## Abstract

**Background:**

Major contributors to AD pathogenesis include aggregates of amyloid‐β (Aβ) peptides, hyperphosphorylated tau protein, and neuroinflammation. No currently approved treatment stops or significantly slows the progression of AD. Nevertheless, one class of agents that has shown promise is metal chelators. For the assessment of a novel effect of oral administration of 1,10‐Phenanthroline‐5‐amine (PAA) on the severity of amyloid plaque load, we used a transgenic (Tg) mouse model with inserted human autosomally dominant (familial) AD genes: amyloid‐beta (Aβ) protein precursor (APP) and tau protein.

**Method:**

APP/Tau transgenic mice that model AD were allotted into one of two groups. The control group received no treatment while the experimental group received 1,10‐phenanthroline‐5‐amine (PAA) in their drinking water, starting at 4 months. All animals were sacrificed at 1 year of age, and their brains were stained with 2 different markers of amyloid plaques, Amylo‐Glo+ and HQ‐O, as we have recently described (Schmued et al, 2023).

**Result:**

PAA administered as a daily oral dose for 9 months resulted in no changes in weight or behavior and resulted in no observed pathologies. Control animals exhibited numerous dense core plaques throughout the neo‐ and allo‐cortical brain regions. PAA administered as a daily dose for 9 months resulted in roughly 2/3 the amyloid plaque burden compared to untreated transgenic mice.

**Conclusion:**

Oral daily dosing with PAA significantly reduced the amyloid plaque burden in transgenic AD model mice. The mode of action of PAA may be attributed to its ability to chelate transition metals and to inhibit either metalloprotease enzymes or the metal‐seeded auto aggregation of Aβ. The underlying mechanism for this protection is not fully known; one possible mechanism would be to inhibit the “metal‐seeding” of Aβ. Dyshomeostasis of certain transition metals in brain microenvironment contributes to amyloidosis. Although this dysregulation may be attributable to impaired metal transporter function, there exists no known treatment that would modify such dysfunction. Further research is underway to confirm these results in another mouse model of AD (APP/PS1). Altogether, reducing regional metal levels via chelation with PAA may be a feasible and viable strategy for suppressing the formation of amyloid plaques in AD patients.

